# COVID-19 and Kidney Disease (KD): A Retrospective Investigation in a Rural Southwestern Missouri Region Patient Population

**DOI:** 10.7759/cureus.41043

**Published:** 2023-06-27

**Authors:** Kailey J Kowalski, Shilpa Bhat, Mariah Fedje, Greg Stahl, Nova Beyersdorfer, Darrin S Goade, Kerry Johnson, Robert Arnce, Robert Hillard

**Affiliations:** 1 College of Medicine, Kansas City University, Joplin, USA; 2 Quality Improvement, Freeman Health System, Joplin, USA; 3 Primary Care, Kansas City University, Joplin, USA; 4 Pharmacy, Freeman Health System, Joplin, USA; 5 Mathematics, Missouri Southern State University, Joplin, USA; 6 Emergency Medicine, Freeman Health System, Joplin, USA; 7 Pathology and Anatomical Sciences, Kansas City University, Joplin, USA

**Keywords:** coronavirus disease 2019 (covid-19), kidney disease (kd), midwest, end stage renal disease (esrd), chronic kidney failure (ckd), acute kidney injury (aki)

## Abstract

Background: Studies have linked pre-existing kidney disease (KD) to higher rates of mortality due to coronavirus disease 2019 (COVID-19) infection. In the rural Midwest, where KD is prevalent, the impact of COVID-19 has been significant in a population that includes many patients on Medicare or Medicaid.

Methods: A retrospective cohort study was performed assessing patients with acute kidney injury (AKI), chronic kidney disease (CKD) and end stage renal disease (ESRD), with and without COVID-19. International Classification of Diseases 10^th^ Revision codes were submitted by physicians into Freeman Health System’s Electronic Medical Records and gathered from April 2020 to January 2021. The data were analyzed and compared to determine whether the mortality rate in patients with varying stages of KD and COVID-19 was higher than the mortality rate in patients with KD alone, excluding variables such as sex and age.

Results: The 95% confidence interval (CI) of the mortality rate of patients with COVID-19 and any degree of KD, encompassing both AKI and CKD, was between 30.21% and 37.63%. This metric was significantly higher than the 95% CI of COVID-19 infection (6.70%-9.96%, p<0.0001) or KD alone (10.89%-13.01%, p<0.0001). Within those with COVID-19 and KD, the highest rate of mortality was in patients with AKI (38.13% and 49.02%). There was not sufficient statistical support in our sample to assert that COVID-19 increased mortality in ESRD patients.

Conclusions: Based on our results, patients with KD and COVID-19 are at higher risk for mortality when compared to patients with KD alone. Further studies are warranted into individual comorbidities affecting KD patient outcomes with COVID-19.

## Introduction

Coronavirus disease 2019 (COVID-19) has been responsible for a global pandemic caused by severe acute respiratory syndrome coronavirus 2 (SARS-CoV-2), a highly infectious respiratory disease with a marked mortality rate [1]. As of December 2021, COVID-19 statistics showed approximately 1.0 million COVID-19 cases and 16,200 deaths in Missouri [2]. 

Throughout the COVID-19 pandemic, studies have looked at risk factors in mortality with pre-existing conditions after infection with SARS-CoV-2. Kidney disease (KD) in particular has been linked to worse outcomes with COVID-19 infection. For example, studies have shown that COVID-19 disproportionately affects people with chronic kidney disease (CKD), with as much as a 10-fold higher incidence of death [3]. Moreover, the poorer renal function a patient has, the greater chance of severe disease with COVID-19 infection -- COVID-19 patients with CKD on dialysis had a shorter time from symptom onset to intensive care unit (ICU) admission and a greater death rate compared to non-dialysis-dependent COVID-19 CKD patients [4]. In the Southwestern Missouri counties containing Joplin and Neosho, CKD prevalence averaged approximately 24% in Medicare beneficiaries -- above average for a given county in the United States [5]. The peak of hospital admissions in the same area for COVID-19 in 2021 was approximately 60 individuals per 100,000 residents [2]. Given this demographic data in the context of the aforementioned studies, a project in this area looking at mortality in relation to COVID-19 infection and CKD is a logical undertaking.

In addition to the above considerations, there is also a high risk of acute kidney injury (AKI), also termed acute renal failure, secondary to COVID-19 infection in patients with no other underlying kidney pathology [6]. As the incidence of both end stage renal disease (ESRD), also termed end stage KD, and dialysis-requiring AKI disproportionally affects the Midwest [7-8], we performed the current study to investigate not simply the outcome of COVID-19 positive patients in CKD, but also their outcome with respect to ESRD and AKI.

Focusing on KD is also important as treating such a disease amounts to a substantial healthcare cost. For instance, in 2019, $87.2 billion was spent on CKD and $37.7 billion on ESRD [9]. Most notably, in rural areas as much as a quarter of the population is enrolled in Medicare, with 22% having dual enrolment in Medicare and Medicaid [10]. Given these facts, it makes sense to focus on a rural population base when looking at COVID-19’s impact on KD. 

For the above reasons, a retrospective analysis was undertaken of COVID-19-positive patients with concomitant KD from a Southwestern Missouri hospital system with a large rural catchment. The study investigates whether COVID-19 with KD results in a higher mortality rate in this population. In addition, it also helps to compare differences in COVID-19 mortality in patients with AKI, CKD, and ESRD.

## Materials and methods

A retrospective observational cohort study was performed in which electronic medical records from the Freeman Health System (Joplin and Neosho, MO) were analyzed based on the International Classification of Diseases 10th Revision (ICD10) codes. We included all adult patients over 18 years of age. The patients are from an area inclusive of Southwestern Missouri and surrounding areas. All ICD10 data from April 1, 2020 through December 31, 2021 were analyzed, which corresponded to 16,894 admitted patients (excluding duplications). Patients who did not have renal failure ICD10 codes listed in Table 1 were excluded. During this timeframe, there were 1,729 patients with the COVID-19 ICD10 code, out of which 625 had renal disease ICD10 codes listed in Table 1. Patient identifiers were removed to maintain patient anonymity and confidentiality. As a retrospective study, consent was not needed. The Institutional Review Board at Freeman Health System approved this study (IRB # 2022001).

**Table 1:** Renal failure/disease ICD-10 codes. The above table contains and defines the International Classification of Diseases 10th Revision (ICD-10) codes used in our inclusion criteria. CKD, chronic kidney disease; ESRD, end stage renal disease

N170	Acute kidney failure with tubular necrosis
N179	Acute kidney failure, unspecified
N181	CKD, stage 1
N182	CKD, stage 2 (mild)
N183	CKD, stage 3 (moderate)
N1830	CKD, stage 3 unspecified
N1831	CKD, stage 3a
N1832	CKD, stage 3b
N184	CKD, stage 4 (severe)
N185	CKD, stage 5
N186	ESRD
N189	CKD, unspecified

**Figure 1:**
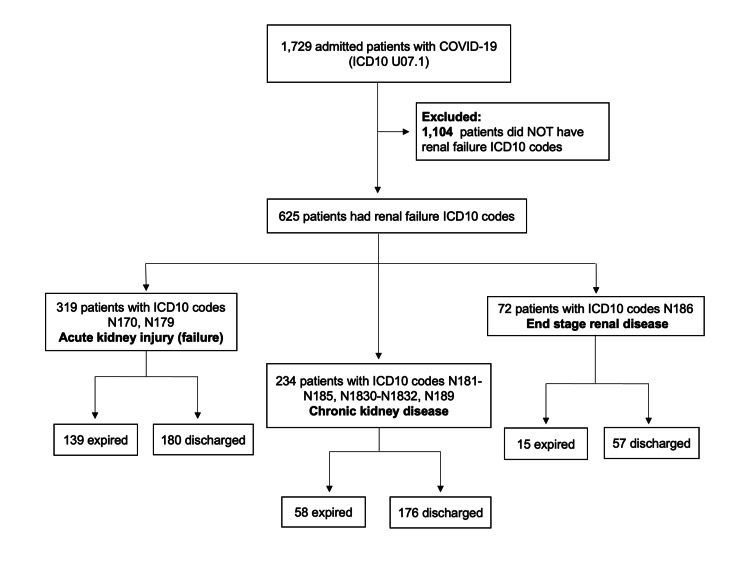
Kidney disease in a COVID-19 patient population. Patients admitted with ICD10 codes for COVID-19 were evaluated for associated ICD10 codes for renal failure. They were then further divided by their degree of renal failure and their final disposition. ICD10, International Classification of Diseases 10th Revision; COVID-19, coronavirus disease of 2019.

**Figure 2:**
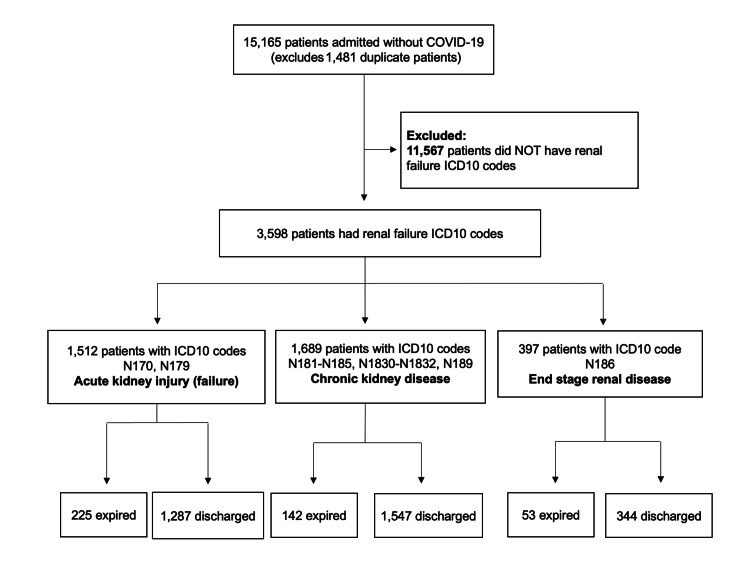
Kidney disease in a COVID-19-negative patient population. Patients admitted without ICD10 codes consistent with COVID-19 were checked for ICD10 codes for renal failure, then further categorized based on their degree of renal failure and their final disposition. ICD10, International Classification of Diseases 10th Revision; COVID-19, coronavirus disease of 2019.

The patients were subsequently grouped by ICD10 code into those with AKI, CKD, and ESRD (Figure 1). Patients without a COVID-19 ICD10 code but having renal disease based on ICD10 codes listed in Table 1, were similarly grouped as controls (Figure 2). 

The mortality rate was determined as the proportion of the group that expired. The data were statistically analyzed using Wald’s method and two sample proportional summary hypothesis tests with confidence intervals for the proportional difference utilizing StatCrunch (web-based statistical software) and Microsoft Excel. The data were considered significant when p<0.05 and a 95% confidence interval (CI) was used.

The study was reported in accordance with the STROBE reporting checklist (the full checklist can be accessed: https://www.strobe-statement.org/checklists/).

## Results

Our sample showed that COVID-19 with any degree of KD (P1) had a mortality rate between 30.21% and 37.63% (95% CI), see Table 2. Using a two-sample proportion test, we found that those in this group (P1) had a mortality between 18.11% and 25.83% higher than those with any KD but COVID-19 negative (P1 vs. P6, p<0.0001) and 21.53%-29.64% higher than those with COVID-19 without any KD (P1 vs. P2, p<0.0001), see Table 3.

**Table 2:** Percentage of mortality and CIs amongst individual populations. Mortality rates among various populations within our sample with 95% CI results. COVID-19, coronavirus disease of 2019; ESRD, end stage renal disease; CKD, chronic kidney disease; AKI, acute kidney injury; CI, confidence interval

Populations	Mortality	Sample proportion	Lower 95% CI	Upper 95% CI
(P1) COVID-19 + any degree of kidney injury/disease	212 of 625	0.3392	0.3021	0.3763
(P2) COVID-19 without any degree of kidney injury/disease	92 of 1104	0.0833	0.0670	0.0996
(P3) COVID-19 + ESRD	15 of 72	0.2083	0.1145	0.3021
(P4) COVID-19 + CKD	58 of 234	0.2479	0.1925	0.3032
(P5) COVID-19 + AKI	139 of 319	0.4357	0.3813	0.4902
(P6) Any degree of kidney injury/disease without COVID-19	430 of 3598	0.1195	0.1089	0.1301
(P7) ESRD without COVID-19	53 of 397	0.1335	0.1000	0.1670
(P8) CKD without COVID-19	142 of 1689	0.0841	0.0708	0.0973
(P9) AKI without COVID-19	225 of 1512	0.1488	0.1309	0.1667

**Table 3:** Comparison of patients with and without COVID-19 and renal failure. Comparison within sample groups of patients with or without COVID-19 and with varying degrees of renal failure. P1 represents patients with COVID-19 and any degree of kidney injury/disease. P2 represents patients with COVID-19 and without any type of kidney injury/disease. P3 are those with COVID-19 and ESRD. P4 are those with COVID-19 and CKD. P5 are those with COVID-19 and AKI. P6 are those without COVID-19 but with some degree of kidney injury/disease. P7 are those without COVID-19 and with ESRD. P8 are those without COVID-19 but with CKD. P9 are those patients without COVID-19 and with AKI. Notably, P3 vs P4, P7 vs P9, and P3 vs P7 had no statistical significance, as their p-values were greater than 0.05. Mortality rates of each population were compared (Sample 1 vs. Sample 2). Upper and lower 95% CIs were calculated as above. S1 = Sample 1; S2 = Sample 2 COVID-19, coronavirus disease of 2019; ESRD, end stage renal disease; CKD, chronic kidney disease; AKI, acute kidney injury; CI, confidence interval

Comparisons	Mortality rate for sample 1	Mortality rate for sample 2	S1 vs. S2	Lower 95% CI for S1-S2	Upper 95% CI for S1-S2	p-value
P1 vs. P2	0.3392	0.0833	0.2559	0.2153	0.2964	<0.0001
P1 vs. P6	0.3392	0.1195	0.2197	0.1811	0.2583	<0.0001
P2 vs. P6	0.0833	0.1195	0.0362	0.0167	0.0556	0.0008
P3 vs. P4	0.2083	0.2479	0.0395	-	-	0.4913
P3 vs. P5	0.2083	0.4375	0.2274	0.1190	0.3358	0.0004
P4 vs. P5	0.2479	0.4375	0.1879	0.1103	0.2655	<0.0001
P7 vs. P8	0.1335	0.0841	0.0494	0.0134	0.0854	0.0023
P7 vs. P9	0.1335	0.1488	0.0153	-	-	0.4416
P8 vs. P9	0.0841	0.1488	0.0647	0.0424	0.0870	<0.0001
P3 vs. P7	0.2083	0.1335	0.0748	-	-	0.0971
P4 vs. P8	0.2479	0.0841	0.1638	0.1069	0.2207	<0.0001
P5 vs. P9	0.4357	0.1488	0.2869	0.2296	0.3442	<0.0001

On looking at the subgroup results, our data do not provide sufficient support to assert that those with COVID-19 and ESRD are at increased risk of mortality compared to ESRD alone (P3 vs. P7) but does show that COVID-19 patients with chronic renal disease have a 10.69%-22.07% higher mortality than non-COVID-19 patients with chronic renal disease (P4 vs. P8, p<0.0001). Similarly, COVID-19 patients with acute renal failure have a 22.96%-34.42% higher mortality than non-COVID patients with acute renal failure (P5 vs. P9, p<0.0001), see Table 3.

**Figure 3:**
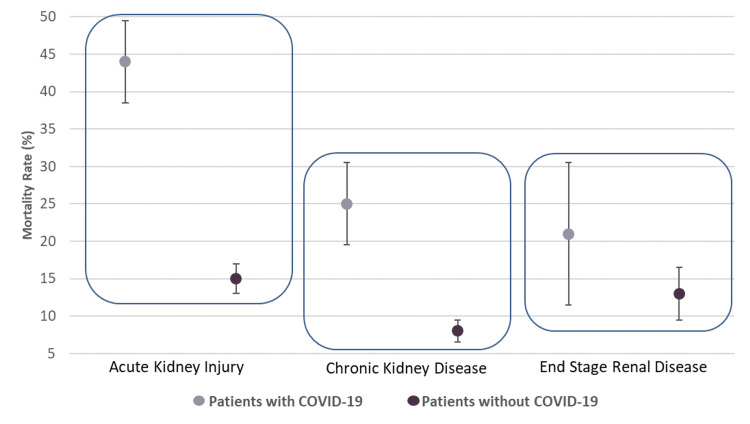
Mortality rates in COVID-19 and renal disease patients. Graph with CIs showing mortality rates in the various major subgroups. COVID-19, coronavirus disease of 2019; CI, confidence interval

Of those with COVID-19 and KD, those with AKI had the highest degree of mortality between 38.13% and 49.02% (p<0.0001), see Table 2. When comparing the mortality rate of patients with COVID-19 and a specific type of renal failure, we found that AKI (P5) showed an 11.9%-33.6% higher mortality rate when compared directly with the ESRD group (P3 vs. P5, p=0.0004) and 11.0%-26.6% higher mortality rate when compared to the CKD group (P4 vs. P5, p<0.001), see Table 3 and Figure 3.

No statistically significant difference was observed between males and females in disease categories, and, when comparing groups by general age (greater or equal to 65 vs. less than 65), mortality trends did not change.

## Discussion

Overall, our data provide a direct causal relationship between COVID-19 infection and increased mortality of patients with renal disease, more specifically AKI and CKD, in a rural Southwestern Missouri hospitalized patient base. Moreover, in those with KD and COVID-19, the mortality is the highest in those with AKI when compared to CKD or ESRD in our study population. These findings are unsurprising, given previous results showing that KD on admission as well as AKI during hospitalization are associated with an increased risk of death in COVID-19 patients [11-12].

There are several factors that explain increased mortality with COVID-19 infection and concomitant AKI. First, COVID-19 patients often have prolonged hypovolemia due to fever (which can cause insensible losses), vomiting, and/or diarrhea. Such prolonged hypovolemia can result in prerenal azotemia compounding any degree of AKI. Second, SARS-CoV-2 is known to attack cells with angiotensin-converting enzyme 2 receptors, precipitating a robust cytokine release [13]. Such a release includes interleukin 6 (IL-6), a proinflammatory factor that increases vascular permeability, ultimately impairing organ function [14]. More specifically in the kidney, IL-6 can contribute to circulatory collapse leading to acute tubular injury and rhabdomyolysis. Furthermore, IL-6 can induce a hypercoagulable state with subsequent thrombotic microangiopathy, further impacting kidney function [13].

Similar to COVID-19 patients with AKI, patients with CKD and concomitant COVID-19 were found to have a significantly higher mortality rate and a poorer prognosis compared to patients with CKD alone, even when excluding those with ESRD requiring renal replacement therapy. Such a worse outcome is thought to be due to increased lymphopenia coupled with neutrophilia and subsequent cytokine storm, which is seen in these patients and is often predicted by a higher initial creatine kinase [15]. Although a worsening in mortality rates of ESRD due to COVID-19 has been documented [15], our results did not show a significant difference in mortality between patients with COVID-19 and concomitant ESRD compared to ESRD alone. This apparent discrepancy may be due to the smaller sample size of ESRD patients in our study population, which may not represent the larger population. Additional data would be helpful to address this issue.

It has been found that rural patients generally have worse COVID-19 outcomes. A study in the Appalachian region suggested that comorbidities such as obesity, negative health-related behaviors such as smoking, and harmful environmental exposures make rural patients more susceptible to COVID-19 [16]. Furthermore, rural regions typically have increased poverty and poorer hospital access which have contributed to COVID-19’s negative effects [17]. Therefore, it is not surprising that rural areas have been especially hard hit in terms of mortality during COVID-19’s initial reach. For example, in Tennessee, mortality was significantly increased in rural areas when compared to urban ones, even after controlling for other variables [18]. Compounding this picture is the fact that individuals in rural areas are at increased risk for the development of CKD -- in agricultural centers in the Midwest, increased rates of CKD are thought to be caused by exposure of nephrotoxic chemicals including fertilizers, herbicides, and pesticides have been documented [19]. Since in rural populations, there is an increased incidence of not only poorer outcomes from COVID-19 but also a greater incidence of KD, the finding that KD coupled with COVID-19 causes increased mortality in a rural Midwest patient population subset is even more significant.

Furthermore, it should be mentioned that vaccine hesitancy is higher in rural populations [20], and since vaccine hesitancy is associated with a general distrust of medical providers [21], this may lead to delayed admission and worse outcomes in these populations. Interestingly, increased mortality can also be seen in highly urban populations with a high degree of Black patients which have a much higher degree of COVID-19 vaccine hesitancy when compared to Whites in the same community [22]. For example, one study performed in one of the nation’s poorest urban counties found that Black patients had the highest odds ratio of developing AKI with COVID-19 (1.7 adjusted odds ratio), and such development was associated with a significantly higher risk of in-hospital death than those with AKI or COVID-10 alone [23]. KD coupled with COVID-19 significantly impacts those on the edge of society whether from an impoverished rural or urban background.

Although our population subset gives important insight, it is also limiting since the demographics of our patient sample are derived from a largely Caucasian, rural, relatively homogenous Midwest catchment area. Therefore, the results are not necessarily generalizable to other regions or ethnicities. Furthermore, this was a retrospective study therefore the sample chosen was not random, and it is unable to be determined if the sample analyzed is representative of the population. Our study is also limited in that concomitant comorbidities that likely play an exacerbating role in the COVID-19 disease process and subsequent mortality (such as diabetes, obesity, or other metabolic disorders) were not considered due to the lack of adequate statistical power in the data set. Therefore, future larger studies of a more diverse patient population that explore the effect of COVID-19 and renal disease in conjunction with other comorbidities should be performed.

## Conclusions

In this study, we found those with COVID-19 and AKI have the greatest mortality when compared to other types of renal injury. COVID-19 was also found to contribute to higher mortality rates in those with CKD than those with CKD alone. Finally, although ESRD showed no significant difference between those with COVID-19 and those without, this may simply be due to the fact that these subgroups are smaller and lack the power to show such differences. 

Although the current study adds to the literature on COVID-19 and renal failure, a further investigation involving the effects of other comorbidities in COVID-19-positive patients with different types of renal failure may be helpful to stratify risk of such patients, and, studies could be performed on a variety of populations. Ultimately, the goal is to arm physicians and healthcare providers with the understanding they need to triage patients to appropriate care levels and affect better outcomes. Such knowledge becomes increasingly crucial as new strains and variants emerge.
